# The Pore Microstructure Evolution and Porous Properties of Large Capillary Pressure Wicks Sintered with Carbonyl Nickel Powder

**DOI:** 10.3390/ma15175830

**Published:** 2022-08-24

**Authors:** Fengshi Zheng, Linshan Wang, Rui Wang, Jianwei Wang, Shaoming Zhang, Qiang Hu, Limin Wang

**Affiliations:** 1 Metal Powder Materials Industrial Technology Research Institute of GRINM, Beijing 101407, China; 2 GRIPM Advanced Materials Co., Ltd., Beijing 101407, China; 3 General Research Institute for Nonferrous Metals, Beijing 100088, China; 4 Beijing GRIPM Advanced Materials Research Institute Co., Ltd., Beijing 101407, China; 5 China Iron & Steel Research Institute Group, Beijing 100081, China

**Keywords:** wicks, carbonyl nickel powder, sintering temperature, maximum pore diameter, permeability

## Abstract

We investigated the effect of different sintering temperatures ranging from 200 °C to 600 °C on the porous properties and pore microstructure of large capillary pressure wicks made of carbonyl nickel powder. The evolution model of hydraulic diameter was established and verified by the maximum pore diameter. Hydraulic diameter changed as the roughness of particle surfaces decreased and sintering necks grew large during sintering. In the contact-formation stage and the initial sintering stage (200–500 °C), the decrease in the roughness of particle surfaces played a decisive role, contributing to an increase in hydraulic diameter. In the intermediate sintering stage (600 °C), the growth of sintering necks dominated the process, however the hydraulic diameter was reduced. These results show that the maximum pore diameter first increased and then decreased in the same way as our evolution model. Permeability and capillary performance of the wicks first increased and then declined with increasing sintering temperature. We found the optimal sintering temperature to be 400 °C, at which point the wicks achieved the maximum pore diameter of 1.21 μm, a permeability of 1.77 × 10^−14^ m^2^, and their highest capillary performance of 1.46 × 10^−8^ m.

## 1. Introduction

Increasingly large heat flux and high heat dissipation makes transferring the heat generated by electronic devices such as central processing units and hard disk drives difficult [1]. Loop heat pipes (LHPs) have attracted attention as one possible solution to this problem due to their long-distance heat transferring capabilities, flexible configurations, various possible working orientations, and ability to transfer heat in systems with only small temperature differences [2,3,4]. The wick inside LHPs, a kind of porous material, is considered the most important component since it provides capillary pressure to circulate the working fluid inside a closed loop where no external power is required, and capillary pressure and permeability are the key parameters of wicks [1]. To optimize their capillary performance, high permeability and powerful capillary pressure are typically needed. However, there is always a tradeoff between permeability and capillary pressure. A smaller pore size increases the capillary pressure of a wick; however, the smaller the pore size is, the lower the permeability is [5]. Considering this tradeoff, wicks are generally divided into two types according to their critical features: high permeability wicks and large capillary pressure wicks. High permeability wicks usually provide LHPs with high heat flux transferring capabilities over a hundred-millimeter distance at horizontal orientation [2,3,6]. Large capillary pressure wicks ensure the efficient operation of LHPs over a thousand-millimeter distance at any orientation [6].

There are several excellent materials for wicks, such as nickel, titanium, polymeric, ceramic, copper and stainless steel, while those made of carbonyl nickel powder possess the highest purity, controllable porosity and pore size, appropriate mechanical properties, moderate thermal conductivity, and compatibility with various working fluids. Hence, carbonyl nickel powder is widely used to fabricate high-performance wicks [6,7,8].

To fabricate high permeability wicks with carbonyl nickel powder, pore forming agents and loose sintering processes are usually adopted, and a comparatively high sintering temperature, which is normally higher than 550 °C, is required to obtain enough strength needed in the assembly and operation of LHP in particular [8,9,10,11]. Qu et al. [12] investigated the effects of sintering temperature and sintering holding time on the thermal performance of bi-porous nickel wicks in LHPs. Their results show that the porosity and permeability of wicks continuously decrease with increasing sintering temperature from 650 °C to 800 °C, and with prolonging sintering holding time from 30 min to 60 min. Mishra et al. [8] developed nickel wicks using the tap-sintering technique and investigated the effects of sintering temperature on the porosity, permeability and mechanical strength of wicks; their results show that the porosity and permeability of wicks decline as sintering temperature increases from 550 °C to 775 °C.

In order to manufacture large capillary pressure wicks, the method of sintering after pressing carbonyl nickel powder without pore forming agents is frequently adopted. Wicks fabricated in this method are praised for their fine pores and high strength, although their porosity and permeability are somewhat low. For LHPs, the maximum pore diameter in wicks is vitally important because there is backflow of vapor from the evaporation zone to the compensation chamber through the pore with the largest diameter due to the inverse dependence of capillary pressure on pore diameter [9]. This kind of wick is usually characterized by a porosity of around 50% and a maximum pore diameter of about 1.0 μm [13,14,15,16,17,18,19]. Although this kind of wick is widely used and exhibits excellent performance, few studies have focused on the fabrication processes of this kind of wick, and its optimal sintering temperature is still unknown.

Sintering processes significantly affect the pore structure and properties of porous materials. In sintering processes, sintering necks form and grow large between particles while porosity and permeability simultaneously decrease [20]. Initially, pores in green porous materials are irregular. As sintering progresses, however, the angular pores form a smooth, near-cylindrical network with little change in pore size [20]. With increasing sintering temperature, the permeability of porous materials usually decreases, and pore size rarely changes or slightly decreases [21,22]. However, early studies have shown that the permeability and pore size of porous materials both increase with sintering temperature [23,24,25,26,27]. Some of these results are attributed to the liquid phase during sintering [25,26,27]. However, other studies have been conducted using the solid-state sintering method, and the reason for the increase in permeability and pore size remains unknown.

Lunin and Kostornov [24] made cylindrical specimens with a porosity of 50% by compacting carbonyl nickel powder, and studied the change in pore diameter distribution while sintering at different temperatures. Their results show that the mean statistical pore diameter first increases and then declines as the sintering temperature increased from 200 °C to 900 °C. Nevertheless, few researchers have studied the change in maximum pore diameter and permeability of porous materials made of carbonyl nickel powder while sintering at different temperatures. In addition, the evolution of the pore size has not been rigorously described.

In this work, we fabricated large capillary pressure wicks with carbonyl nickel powder by cold isostatic pressing followed by sintering, and studied the effects of sintering temperature on their hydraulic diameter, permeability and capillary performance. In order to analyze the changes in permeability and hydraulic diameter, the porosity and volume-specific surface area were determined, and we then investigated the fracture morphology and cross-section morphology of the wicks. Additionally, we established an evolution model of hydraulic diameter and validated it using the parameter of maximum pore diameter. This work promotes understanding of the increase in pore size and permeability during sintering carbonyl nickel powder, and also provides insight into methods of fabrication for large capillary pressure wicks at an optimal sintering temperature.

## 2. Materials and Methods

**Figure 1:**
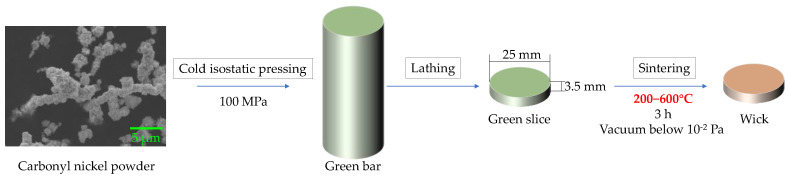
Experimental procedure.

Commercial carbonyl nickel powder with a mean diameter of 1.78 μm was chosen as the raw material, and its bulk density was 0.35 $g\cdot {\mathrm{cm}}^{-3}$. Figure 1 shows the experimental procedure in this study. Uniform green bars were first fabricated by cold isostatic pressing at 100 MPa. Then, the green bars were cut into green slices with a diameter of 25 mm and a thickness of 3.5 mm on a lathe. Finally, the green slices were sintered in a tube furnace, and the wicks were prepared. The sintering process was conducted for 3 h in a vacuum below $1.0\;\times \;10^{-2}\;\mathrm{Pa}$. The sintering temperatures ranged from 200 °C to 600 °C to investigate the effects of sintering temperature on the properties of wicks, and this temperature range was chosen in order to avoid large sintering shrinkage and extreme porosity decrease.

Permeability of green slices and wicks was measured and used together with maximum pore diameter to calculate capillary performance. Digital pressure gauges with an accuracy of ±1 kPa and mass flow controllers with an accuracy of ±2.5 mL·min^−1^ were used to determine the permeability according to ISO 4022: 2018, where nitrogen passed through samples at flow rates ranging from 50 mL·min^−1^ to 500 mL·min^−1^. Absolute alcohol, nitrogen and a digital pressure gauge with an accuracy of ±1 kPa were used to determine the bubble point pressure $P_{b}$ of samples according to ISO 4003: 1977. The maximum pore diameter $d_{max}$ is calculated by Equation (1):

(1) $$d_{max}=\frac{4\;\cdot \;\cos\theta \;\cdot \;\gamma }{P_{b}}$$

where θ is the contact angle between absolute alcohol and samples, and γ is the surface tension of absolute alcohol. Radial sintering shrinkage ratio, porosity and specific surface area were measured to investigate changes in permeability. The radial sintering shrinkage ratio $\frac{∆L}{L_{0}}$ is calculated by Equation (2):

(2) $$\frac{∆L}{L_{0}}=\frac{L_{0}-{\;L}_{1}}{L_{0}}\;\times \;100\%$$

where $L_{0}$ is the diameter of a green slice, and $L_{1}$ is the diameter of the corresponding wick. The diameters of green slices and corresponding wicks were measured using a vernier caliper with an accuracy of ±0.01 mm. According to ISO 2738: 1999, an analytical balance with an accuracy of ±0.0001 g was used to determine the mass of samples m and to determine the volume of samples V by weighing the samples coated with petroleum jelly both in the air and in the water. The porosity of samples is calculated by Equation (3):

(3) $$\varepsilon =(1\;-\frac{m}{V\cdot \rho })\;\times \;100\%$$

where ρ is the true density of nickel. The surface area of samples was determined by the Brunauer–Emmett–Teller (BET) nitrogen adsorption method (Tristar plus 3030, Micromeritics Instruments Corporation, Norcross, GA, USA) according to ISO 9277: 2010, and the volume-specific surface area was calculated by dividing surface area by the volume of samples V. At each sintering condition, 5 wicks were prepared and tested to determine these properties and characteristics. In addition, three samples with fractured surfaces and cross-sections of pores were prepared and observed using scanning electron microscopy (SEM, JSM-F100, JEOL Ltd., Tokyo, Japan).

The uncertainties for measurement of the permeability, bubble point pressure, diameter, mass and volume were estimated to be 0.05 × 10^−14^ m^2^, 1.8 kPa, 0.04 mm, 0.005 g and 10 mm^3^, respectively. In addition, the relative uncertainty for measurement of the surface area was 5%. Using the standard error analysis method, the uncertainties of maximum pore diameter and radial sintering shrinkage ratio were 0.02 μm and 0.3%, respectively, and those of porosity and volume-specific surface area were within 0.6% and 0.18 × 10^6^ m^−1^, respectively.

## 3. Results and Discussion

### 3.1. Evolution of Porosity and Volume-Specific Surface Area

High permeability enhances the heat transfer limit of LHPs because it reduces pressure drop of fluids while filtering through wicks during the operation of LHPs [9]. The permeability K is related to the porosity ε and volume-specific surface area S by the Kozeny–Carman equation:

(4) $$K=\frac{\varepsilon^{3}}{C\cdot S^{2}}$$

where C is an empirical constant that depends on the material and fabrication process. Hence, porosity and volume-specific surface area are important characteristics for wick performance.

#### 3.1.1. Porosity

**Figure 2:**
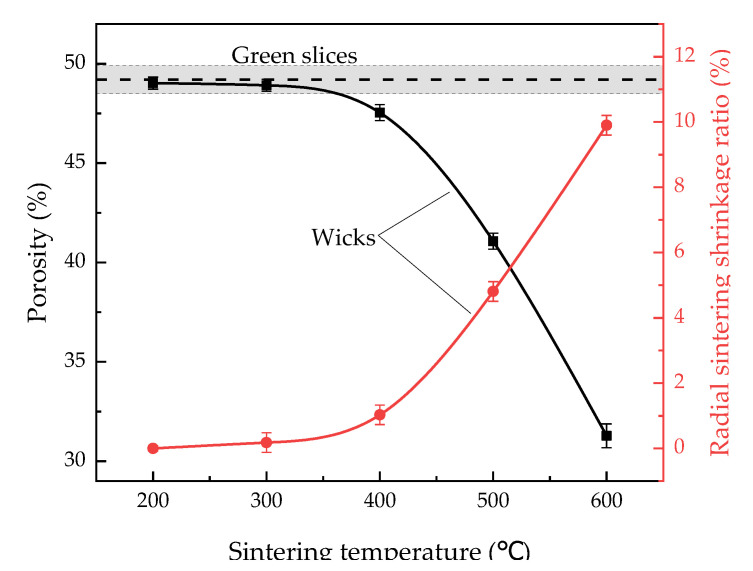
Radial shrinkage and porosity of green slices and wicks sintered at different temperatures.

Figure 2 shows the porosity and radial sintering shrinkage ratio of green slices and wicks sintered at different temperatures. As we can see, the radial sintering shrinkage ratio increased while the porosity decreased with increasing sintering temperature. A large sintering shrinkage ratio of a wick meant great reductions in both its dimensions and volume in sintering, which caused a huge decrease in its porosity, considering its mass remains unchanged in sintering. Related to the small radial sintering shrinkage at 200 °C and 300 °C, the porosity of wicks showed little change compared to the porosity of the green slices, which was 49.2%. When the wicks were sintered at 400 °C, the radial shrinkage ratio slightly increased to 1.0%, hence the porosity slightly decreased to 47.5%. As the sintering temperature rose to 500 °C, the radial sintering shrinkage of the wicks evidently grew as well, so their porosity sharply dropped. At 600 °C, the radial sintering shrinkage ratio of wicks increased to 9.9%, and thus the porosity declined to 31.3%.

Grain-boundary diffusion dominates in the sintering shrinkage of wicks in the presence of low temperatures and small particle diameters [28]. The sintering shrinkage of the wicks in our case indicates that the grain-boundary diffusion of carbonyl nickel powder with a mean diameter of 1.78 μm commences at 400 °C.

#### 3.1.2. Volume-Specific Surface Area

**Figure 3:**
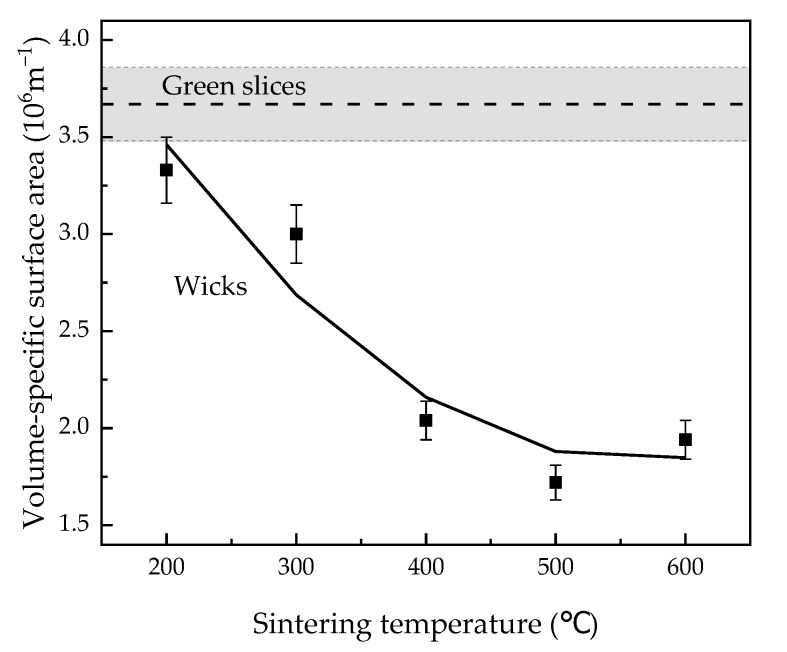
Volume-specific surface area of green slices and wicks sintered at different temperatures.

Figure 3 shows the volume-specific surface area of green slices and wicks sintered at different temperatures. With increasing sintering temperature, the volume-specific surface area declined rapidly at 200–400 °C and slowly at 400–600 °C. When the wicks were sintered at 200 °C, the volume-specific surface area declined only slightly to $3.33\;\times \;10^{6}{\;m}^{-1}$ compared to that of the green slices, which was $3.67\;\times \;10^{6}{\;m}^{-1}$. At 400 °C, the volume-specific surface area significantly decreased to $2.04\;\times \;10^{6}{\;m}^{-1}$. With a further increase in sintering temperature, once again only slight declines were detected in the volume-specific surface area.

**Figure 4:**
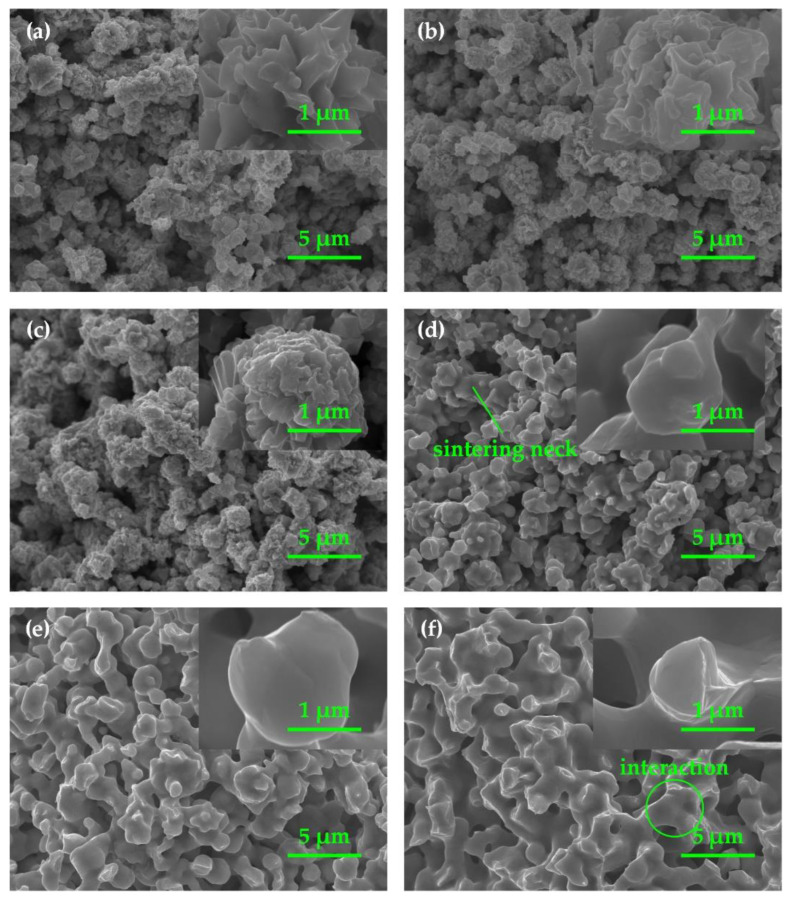
Fracture morphology of green slices and wicks sintered at different temperatures: (**a**) green slice, (**b**) 200 °C, (**c**) 300 °C, (**d**) 400 °C, (**e**) 500 °C, (**f**) 600 °C.

To study the change in volume-specific surface area, we observed the microstructure of green slices and wicks by SEM. Figure 4 shows the fracture morphology of a green slice and various wicks sintered at temperatures between 200 °C and 600 °C. As we can see, higher sintering temperatures led to smoother surfaces of particles and larger sintering necks between particles. Rough particle surfaces were detected in the green slice, but the increase in sintering temperature from 200 °C to 600 °C gradually smoothed the surfaces of the particles. The most pronounced decline in roughness of the particle surfaces is found at 200–400 °C.

Moreover, clear sintering necks formed between particles at 400 °C and grew larger with increasing sintering temperatures. Sintering is divided into four stages, which are the contact-formation stage where few sintering necks form, the initial stage where sintering necks grow without interaction with neighboring ones, the intermediate stage where sintering necks interact with neighboring ones, and the final stage where tubular pores pinch closed to form discrete spherical or lenticular pores [20]. As shown in Figure 4, few sintering necks were detected in wicks sintered at 200 °C and 300 °C; sintering necks formed and grew without interaction with neighboring ones at 400 °C and 500 °C; sintering necks interact with neighboring ones at 600 °C. Hence, sintering at 200 °C and 300 °C stopped in the contact-formation stage; sintering at 400 °C and 500 °C stopped in the initial stage; and sintering at 600 °C stopped in the intermediate stage.

Surface diffusion causes the decrease in roughness of particle surfaces, and grain-boundary diffusion together with surface diffusion contributes to the formation and growth of sintering necks. In the sintering process, atoms on rough surfaces move from a convex point to a concave point due to the chemical potential gradient introduced by the curvature gradient, leading to the decrease in roughness of particle surfaces [29]. Moreover, the final shape of the particles depends on the surface diffusion coefficient, which increases with sintering temperature [30]. As the sintering temperature increases from 200 °C to 600 °C, more atoms disperse away from convex points and accumulate at concave points due to the increasing surface diffusion coefficient. As a result, higher sintering temperatures result in smoother particle surfaces and larger sintering necks.

Grain-boundary diffusion also contributes to the growth of sintering necks. Vacant lattice points diffuse away from sintering necks and sink at the grain boundary, which results in sintering shrinkage and neck growth [31,32]. As sintering temperature increases from 400 °C to 600 °C, the grain-boundary diffusion accelerates, leading to increased sintering shrinkage and growth of sintering necks.

The decrease in roughness of particle surfaces and the growth of sintering necks together contribute to the decline in volume-specific surface area (Figure 3). The most pronounced decline in the roughness of the particle surfaces induces the volume-specific surface area to decrease significantly at 200–400 °C. Furthermore, the growth of sintering necks causes the slow decline in volume-specific surface area at 400–600 °C.

### 3.2. Evolution of Pore Size

#### 3.2.1. Evolution of Mean Pore Diameter

For a unique pore with an irregularly shaped cross section, the hydraulic diameter $d_{h}$ of the pore at any of its cross sections is defined in Equation (5) [33] as:

(5) $$d_{h}=4\cdot \frac{A_{pore}}{P_{pore}}$$

where $A_{pore}$ is the area of the pore at the cross section, and $P_{pore}$ is the perimeter. Countless through pores exist in porous materials, and the hydraulic diameter of every through pore varies at different cross sections. By analyzing cross-sectional images of green slices and wicks, we determined the mean pore diameter that is capable of reflecting the size evolution of pores. The uncertainties for measurement of the area and perimeter were estimated to be 0.019 μm^2^ and 0.010 μm, respectively. Using the standard error analysis method, the uncertainty of the mean pore diameter was within 0.015 μm.

**Figure 5:**
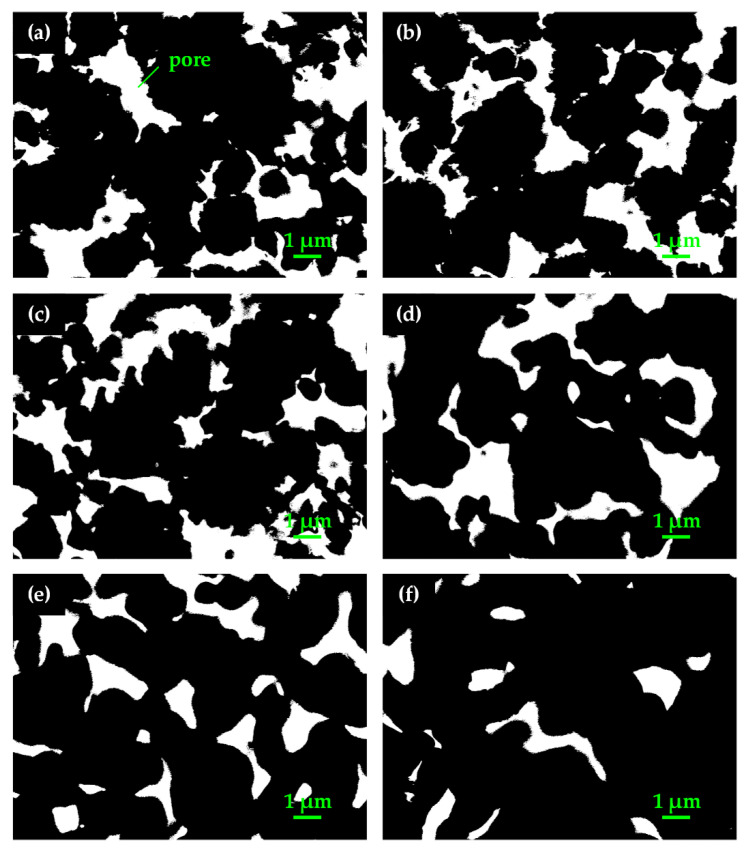
Cross-section morphology of pores in green slices and wicks sintered at various temperatures: (**a**) green slice, (**b**) 200 °C, (**c**) 300 °C, (**d**) 400 °C, (**e**) 500 °C, (**f**) 600 °C.

**Figure 6:**
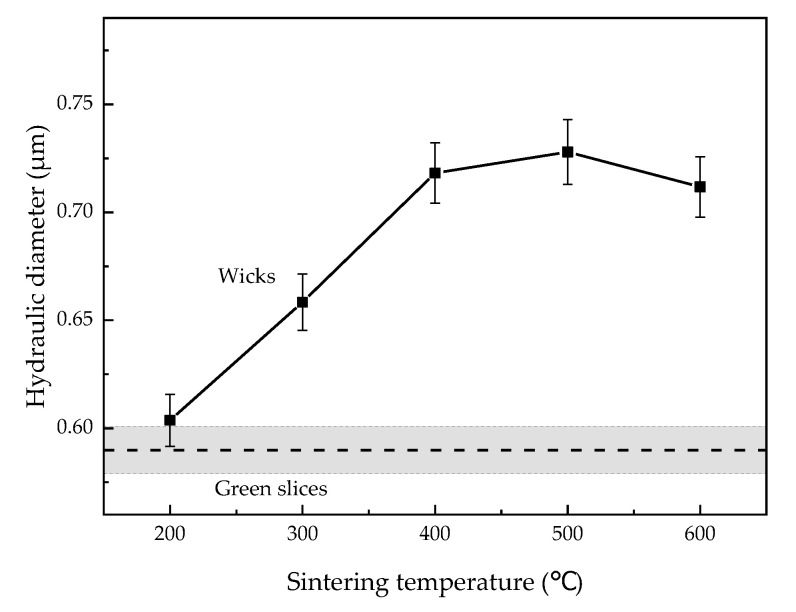
Mean pore diameter of green slices and wicks sintered at different temperatures.

Figure 5 shows the cross-section morphology of pores in green slices and wicks sintered at temperatures from 200 °C to 600 °C. The mean pore diameter of the green slices and wicks was determined from cross-sectional images and is shown in Figure 6. As seen in Figure 5a, the proportion of areas covered by pores is large, and the outlines of pores are zigzag in the green slice. With increasing sintering temperature, the proportional area of pores decreased, and the outlines gradually became smooth as shown in Figure 5b–f. From Figure 6, we see that the mean pore diameter first increased to the maximum at 500 °C, and then declined with an increase in sintering temperature.

The decrease in the roughness of particle surfaces (Figure 4a–f) smoothed the outlines of the pores at the cross section. With increasing sintering temperature, the roughness of particle surfaces gradually decreased, so the outlines of pores gradually became smoother. The growth of sintering necks (Figure 4d–f) reduced the proportional volume of pores in wicks. Hence, the perimeter and area of every single pore and the proportional area of pores declined at the cross section. As shown in Figure 6, the wicks sintered at 200 °C and the green slices were almost the same in terms of mean pore diameter, 0.604 μm and 0.590 μm, respectively. When the wicks were sintered at 300 °C, the roughness of the particle surfaces decreased while sintering necks formed. As a result, the perimeter of the pores declined, and the area showed little variation, resulting in an increase in the mean pore diameter.

At sintering temperatures of 400 °C and 500 °C, the roughness of particle surfaces decreased significantly, and sintering necks formed and gradually grew. Additionally, the perimeter of the pores dropped faster than the area, so the mean pore diameter increased to 0.728 μm at the sintering temperature of 500 °C. As the sintering temperature further increased to 600 °C, the particle surfaces showed little change, but the sintering necks grew rapidly. Greater reduction was found in the area of pores compared to the perimeter, leading to the decline in the mean pore diameter. The change in the mean pore diameter at 600 °C indicates that the growth of sintering necks caused more reduction in the area of pores than in their perimeter.

**Figure 7:**
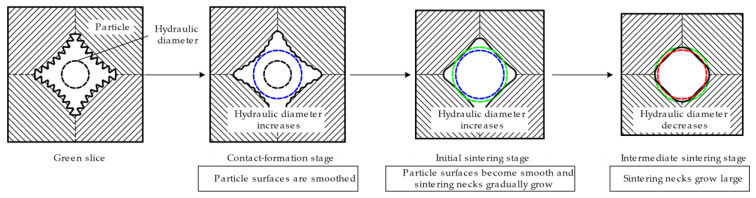
Evolution model of hydraulic diameter in different sintering stages.

Figure 7 summarizes the evolution of the hydraulic diameter in different sintering stages. When the wicks are sintered in the contact-formation stage (200–300 °C) and the initial sintering stage (400–500 °C), the decrease in the roughness of particle surfaces played a decisive role, contributing to an increase in hydraulic diameter. In the intermediate sintering stage (600 °C), the growth of sintering necks dominated the process, however reduced the hydraulic diameter.

#### 3.2.2. Evolution of Maximum Pore Diameter

**Figure 8:**
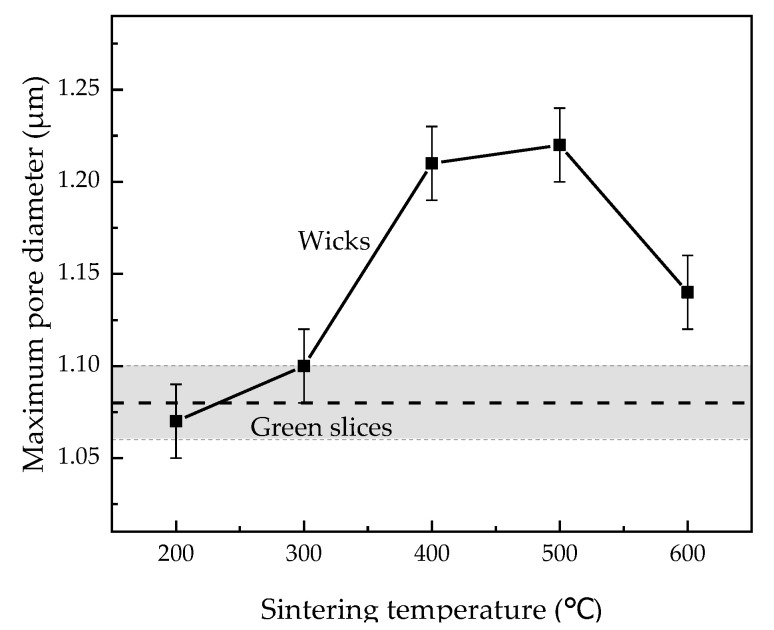
Maximum pore diameter of green slices and wicks sintered at different temperatures.

Maximum pore diameter is defined as the maximum among all the hydraulic diameters of the most constricted part in every through pore [33]. The study of maximum pore diameter is of great importance because the failures of LHPs usually commence at the pore with the largest diameter [9]. Figure 8 plots the maximum pore diameter of green slices and wicks sintered at different temperatures. As the sintering temperature increased from 200 °C to 600 °C, the maximum pore diameter first increased and then decreased in the same way as the evolution model of the hydraulic diameter (Figure 7). The wicks sintered at 400 °C and 500 °C are nearly equal in maximum pore diameter, at 1.21 μm and 1.22 μm, respectively. The experimental evolution of maximum pore diameter thus validates the evolution model of the hydraulic diameter, and this model may be applied to the pore size evolution during solid-state sintering for other particles with rough surfaces. As seen in Figure 8, sintering at 300–600 °C always enlarges the maximum pore diameter of wicks, which is unsatisfactory. However, a sintering process is still needed to enhance the permeability and capillary performance of wicks.

### 3.3. Porous Properties

#### 3.3.1. Permeability

**Figure 9:**
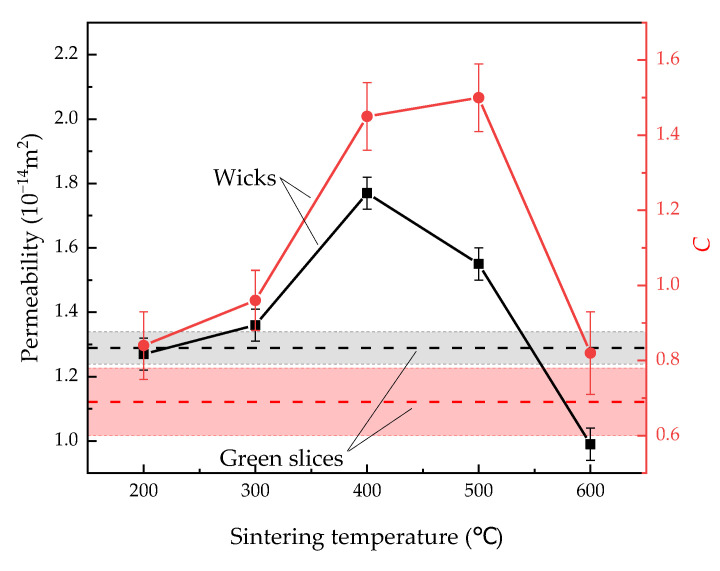
Permeability and *C* of green slices and wicks sintered at different temperatures.

Figure 9 shows the permeability and C of green slices and wicks sintered at different temperatures. With increasing sintering temperatures, the permeability of the wicks first reached the peak value of 1.77 × 10^−14^ m^2^ at 400 °C, and then decreased. When the wicks were sintered at 200 °C, the volume-specific surface area (Figure 3) and the porosity (Figure 2) barely changed, resulting in similar permeability compared to the green slices. From Equation (4) and observed values of porosity, volume-specific surface area and permeability, the empirical constant C was calculated, and the uncertainty of C was within 0.11. As sintering temperature increased from 200 °C to 600 °C, C first increased and then decreased. Wicks sintered at 400 °C and 500 °C showed nearly the same values of C, which were 1.45 and 1.50, respectively.

#### 3.3.2. Capillary Performance

**Figure 10:**
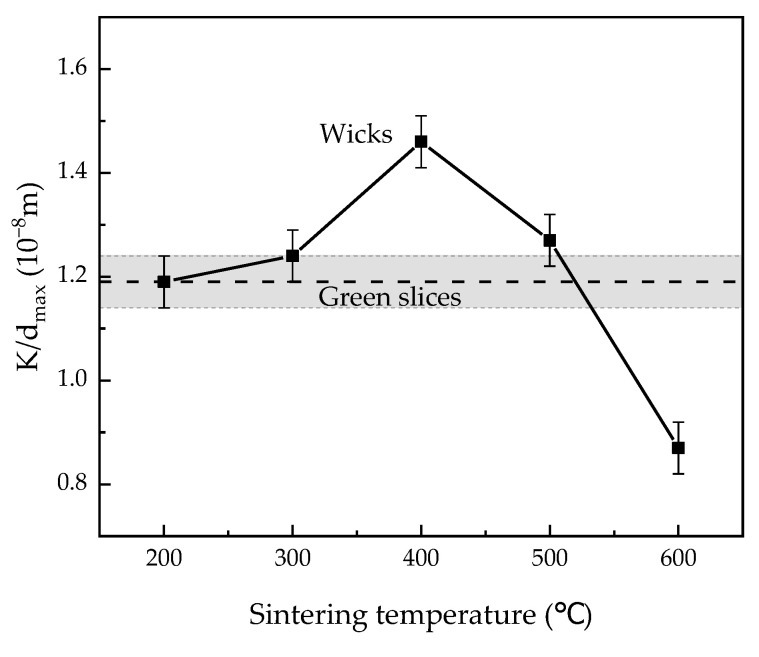
Capillary performance of green slices and wicks sintered at different temperatures.

Capillary performance parameter is usually defined as the ratio of permeability to pore diameter [34,35]. In this study, we use the ratio of permeability to the maximum pore diameter ${K/d}_{max}$ to evaluate the capillary performance of our wicks because the maximum diameter of the wicks determines the heat transfer limit of LHPs [9]. Figure 10 shows the capillary performance of green slices and wicks sintered at different temperatures, and the uncertainty of ${K/d}_{max}$ was 0.05 × 10^−8^ m. The capillary performance first increased to the maximum at 400 °C, and then declined with increasing sintering temperatures. The changes in the capillary performance of the wicks show that the sintering temperature of 400 °C granted the wicks the highest capillary performance of 1.46 × 10^−8^ m. When the wicks were sintered at 400 °C, the capillary performance and the permeability increased by 23.7% and 37.5%, respectively, while the maximum pore diameter increased by 12.0% compared to the green slices.

## 4. Conclusions

Based on the pore microstructure and porous properties of large capillary pressure wicks sintered at different temperatures, a summary is proposed:

(1) The pore size first increased and then decreased with increasing sintering temperature from 200 °C to 600 °C. When the wicks were sintered at 400 and 500, the maximum pore diameter reached 1.21 μm and 1.22 μm, respectively, which were the highest levels in this study.
(2) The evolution model of hydraulic diameter with increasing sintering temperature was established and validated. In the contact-formation stage and the initial sintering stage (200–500 °C), the decrease in the roughness of particle surfaces played a decisive role. More reduction in the perimeter compared to the area led to an increase in hydraulic diameter. In the intermediate sintering stage (600 °C), the growth of sintering necks took on a dominant role, which caused more reduction in the area than in the perimeter, leading to a decline in hydraulic diameter.
(3) The optimal sintering temperature proved to be 400 °C, at which point the wicks achieved the permeability of 1.77 × 10^−14^ m^2^ and their highest capillary performance of 1.46 × 10^−8^ m.

## Data Availability

Not applicable.
